# P-2059. Perceptions and Factors Associated with Uptake of Monovalent XBB.1.5 BNT162b2 COVID-19 Vaccine among Healthcare Workers in Peru

**DOI:** 10.1093/ofid/ofae631.2215

**Published:** 2025-01-29

**Authors:** Jose A Gonzales-Zamora, Julieta M Araoz-Salinas, Carlos Quispe-Vicuña, Martín Ernesto Reátegui García, Brando Ortiz-Saavedra, Anderson N Soriano-Moreno, Hans Baltazar-Ñahui, Jorge Alave

**Affiliations:** Infectious Disease Division. University of Miami, Miller School of Medicine., Miami, Florida; Peruvian American Medical Society, Lima, Lima, Peru; Red de Eficacia Clínica y Sanitaria (REDECS), Lima, Perú, Lima, Lima, Peru; Universidad Nacional de la Amazonia Peruana, Iquitos, Peru, Iquitos, Loreto, Peru; Universidad Nacional de San Agustín de Arequipa, Arequipa, Arequipa, Peru; Universidad Peruana Unión, Lima, Lima, Peru; Sociedad Científica de San Fernando, Universidad Nacional Mayor de San Marcos, Lima, Lima, Peru; Universidad Peruana Union, Lima, Lima, Peru

## Abstract

**Background:**

The implementation of the XBB.1.5 BNT162b2 COVID-19 vaccine (updated vaccine) occurred in Peru in January 2024, since then, many questions about its efficacy and safety have been raised. Addressing these concerns is crucial, especially among healthcare workers, given their high risk of acquiring COVID-19 and their role in promoting vaccination. This study investigated the factors and perceptions associated with healthcare workers’ uptake of the updated vaccine.

**Figure 1:**
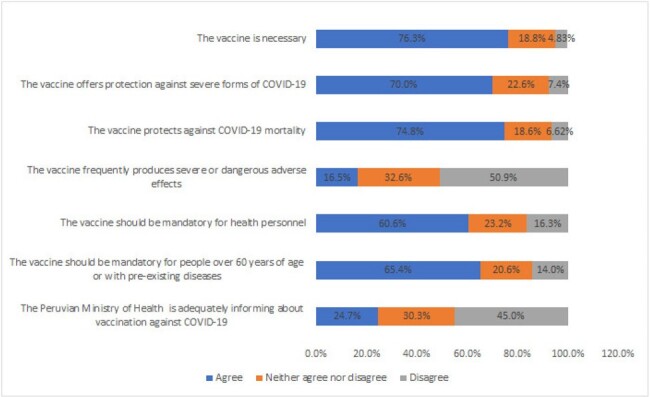
Perceptions about the updated monovalent vaccine among healthcare workers in Peru

**Methods:**

We conducted a cross-sectional study based on an online survey from March 21 to May 1, 2024. Healthcare workers (physicians, nurses, etc.) living and practicing in Peru were included. We calculated crude prevalence ratios with 95% confidence intervals to determine the associated factors and perceptions using Poisson regression with robust variance.

**Table 1:**
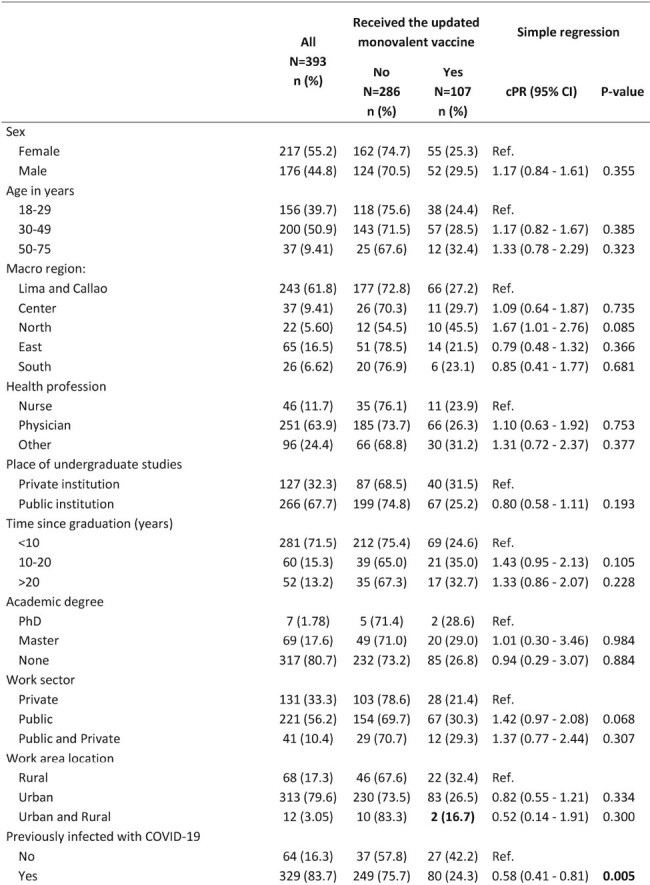
Factors associated with uptake of the updated monovalent vaccine among healthcare workers (Page 1)

**Results:**

We included 393 healthcare workers, predominantly female (55.2%) and physicians (63.9%), almost half aged 30-49 years (50.9%). The majority (72.8%) reported not being vaccinated with the updated vaccine, but 65% of them intended to do it. Regarding perceptions, more than 70% of respondents thought the updated vaccine was necessary and protective against severe COVID-19 and mortality, but only 16.5% believed it caused severe adverse events. Only 24.7% felt the Peruvian Ministry of Health was informing well about the vaccines (Figure 1). In the bivariate analysis, the uptake of this vaccine was significantly higher among those who had a history of COVID-19 (p = 0.005), had received the bivalent vaccine (p< 0.001), or more than five vaccine doses (p< 0.001), and among those who felt the vaccine should be recommended (p=0.015) or even mandatory for high-risk people (p=0.04). On the other hand, the uptake was lower in those who thought the vaccine was not necessary (p=0.043) or did not protect against mortality (p=0.037) (Table 1).

**Table 1:**
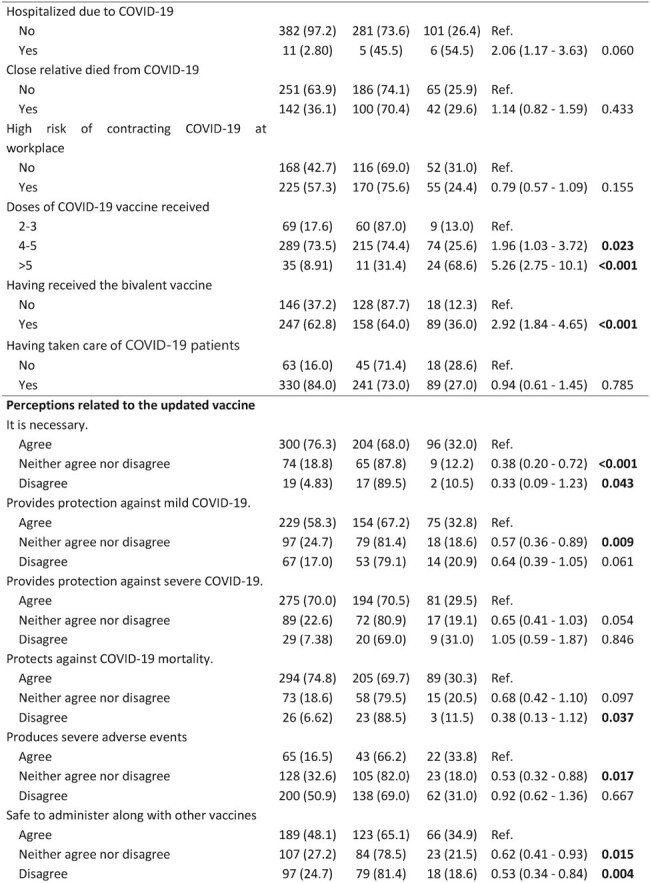
Factors associated with uptake of the updated monovalent vaccine among healthcare workers (Page 2)

**Conclusion:**

History of COVID-19, receiving multiple vaccine doses, and recommending the updated vaccine for high-risk people are factors associated with vaccine uptake. There are still major concerns about the government's communication regarding vaccination. These findings underscore the importance of targeted interventions and communication strategies to improve the poor vaccination rate among healthcare workers in Peru.

**Table 1:**
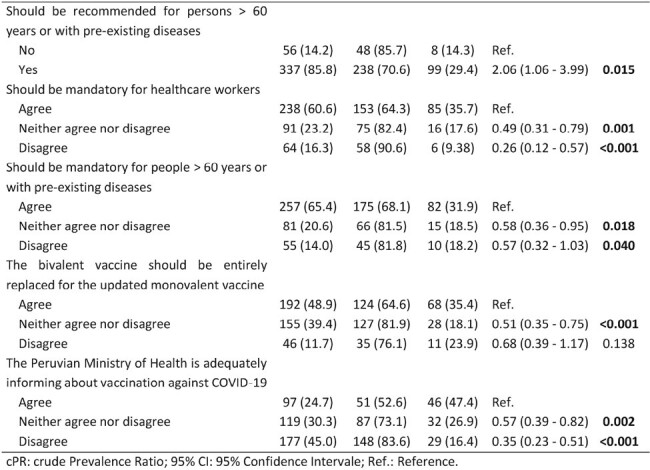
Factors associated with uptake of the updated monovalent vaccine among healthcare workers (Page 3)

**Disclosures:**

All Authors: No reported disclosures

